# Serum Homocysteine Levels and All-Cause and Cause-Specific Mortality in Korean Adult Men: A Cohort Study

**DOI:** 10.3390/nu16162759

**Published:** 2024-08-19

**Authors:** Minyoung Kim, Sujeong Shin, Eunsol Yoo, Jae-Heon Kang, Eunju Sung, Cheol-Hwan Kim, Hocheol Shin, Mi Yeon Lee

**Affiliations:** 1 Department of Family Medicine, Kangbuk Samsung Hospital, Sungkyunkwan University School of Medicine, Seoul 03181, Republic of Korea; s2solar@naver.com (M.K.);; 2 Division of Biostatistics, Department of R&D Management, Kangbuk Samsung Hospital, Sungkyunkwan University School of Medicine, Seoul 03181, Republic of Korea

**Keywords:** homocysteine, mortality, cardiovascular disease, vitamin, cohort study

## Abstract

Background: Hyperhomocysteinemia can increase the risk of cardiovascular disease (CVD), cancer, and neurological disorders; however, hypohomocysteinemia is generally not considered harmful. This study aimed to evaluate the relationship between all levels of homocysteine, both low and high homocysteine levels, and the risk of all-cause and cause-specific mortality in adult Korean men. Methods: Adult Korean men (*n* = 221,356) were categorized into quintiles based on their homocysteine levels. The primary endpoints were all-cause, CVD, cancer, and dementia mortality. Hazard ratios were calculated using Cox proportional hazards models, and the dose–response relationship between homocysteine levels and mortality risk was further explored using restricted cubic spline models. Results: Compared with the reference category (Q2, 8.8–9.9 µmol/L), there was a significant increase in all-cause mortality associated with both low and high levels after multivariable adjustment (*P*_interaction_ = 0.002). Additionally, in spline regression, a U-shaped association between homocysteine levels and all-cause and CVD mortality was observed (inflection point = 9.1 µmol/L). This association was not observed in the vitamin supplementation subgroup. Conclusion: Among Korean adult men, both low and high homocysteine levels increased the risk of all-cause and CVD mortality, indicating a U-shaped relationship. However, this relationship was not statistically significant with vitamin supplementation, suggesting a potential protective role for vitamins.

## 1. Introduction

Homocysteine, an intermediate metabolite derived from dietary methionine, is involved in two crucial biochemical pathways. It can be converted into S-adenosylmethionine, which plays a pivotal role in DNA expression by transferring methyl groups, or it can be utilized to produce glutathione, contributing to its antioxidant capabilities [1,2].

However, if the serum homocysteine level increases without these two metabolic processes, it may indicate hyperhomocysteinemia, a condition characterized by homocysteine levels greater than 15 µmol/L in the blood [3]. This condition is associated with a wide range of diseases. It can increase the risk of cardiovascular disease (CVD), cancer, and dementia [4,5,6]. Hyperhomocysteinemia was also associated with increased all-cause mortality, including in patients with dementia [7,8], and a recent study on Korean populations demonstrated a correlation between increased homocysteine levels and increased mortality from all causes, CVD, and cancer [9]. Additionally, it is known that for every 5 umol/L increase in serum homocysteine, all-cause mortality increases by 33.6% [8].

Although much research on homocysteine has focused on its high levels, recent studies have suggested that low levels may also pose significant health risks. Research shows that cancer patients have lower serum homocysteine levels (7.6 µmol/L) than non-cancer patients by 0.898 times [10]. Additionally, low homocysteine levels were associated with idiopathic peripheral neuropathy (<6 µmol/L) [11], increased Alzheimer’s dementia incidence rates (≤8.9 µmol/L), [12] and increased mortality risks in elderly individuals aged <75 years with severe cognitive impairment (<9.3 µmol/L) [13]. However, most studies have targeted non-Asian populations and included women whose serum homocysteine levels can vary due to the menstrual cycle, pregnancy, and menopause. These hormone-driven variations may confound studies on homocysteine levels and mortality [14,15,16].

Therefore, we aimed to evaluate the association between low and high homocysteine levels and the risk of all-cause and cause-specific mortality in a large cohort of adult Korean men.

## 2. Materials and Methods

### 2.1. Study Population 

**Figure 1:**
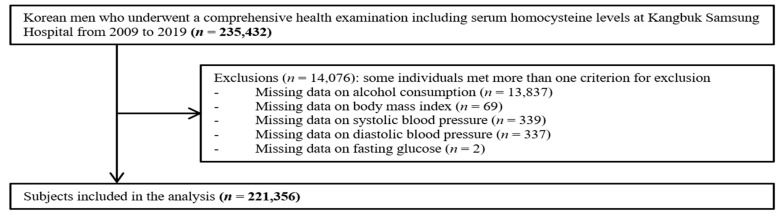
Flow chart of study subjects.

The study subjects were subjects of the Kangbuk Samsung Health Study, a cohort study involving Korean men and women aged ≥18 years who underwent comprehensive health examinations at Kangbuk Samsung Hospital Total Healthcare Centers in Seoul and Suwon, South Korea. This cohort included all men aged ≥18 years who participated in screenings, including homocysteine levels, from 2 January 2009 to 31 December 2019 (*n* = 235,432). We excluded 14,076 participants because of missing data on alcohol consumption, body mass index (BMI), blood pressure, or fasting glucose. Because some individuals met more than one exclusion criterion, the total number of eligible participants was 221,356 (Figure 1). The study protocol was approved by the Institutional Review Board of Kangbuk Samsung Hospital (No: KBSMC 2023-10-022), which waived the requirement for informed consent because we used pre-existing de-identified data obtained during health screening examinations.

### 2.2. Measurements

Self-administered standardized questionnaires were used to identify sociodemographic characteristics, medical history, medication and vitamin supplement use, smoking status, alcohol intake, and exercise frequency [17,18]. Smoking status was categorized as never smoker, former smoker, or current smoker. Alcohol consumption was categorized as <20 or ≥20 g/day. We also evaluated the weekly frequency of moderate- and vigorous-intensity physical activities. Education level was categorized as less than college education, college education, or higher.

Trained nurses measured height, weight, and blood pressure. Hypertension was defined as a systolic blood pressure ≥140 mmHg, diastolic blood pressure ≥90 mmHg [19], self-reported history of hypertension, or current use of antihypertensive medication. Diabetes mellitus was defined as a fasting serum glucose level of ≥126 mg/dL [20] or the use of insulin or glucose-lowering medication. Dyslipidemia was defined as the use of lipid-lowering medications. Vitamin use was defined as the use of multivitamins; folic acid; or vitamins B, C, D, or E.

Blood specimens were obtained from the antecubital vein after >10 h of fasting. Fasting blood measurements included creatinine, total cholesterol, low-density lipoprotein cholesterol (LDL-C), high-density lipoprotein cholesterol (HDL-C), triglycerides, glucose, glycated hemoglobin (HbA1c), insulin, and high-sensitivity C-reactive protein (hsCRP) levels. The estimated glomerular filtration rate (eGFR) was calculated using the Chronic Kidney Disease Epidemiology Collaboration (CKD-EPI) creatinine equation [21]. We calculated the homeostasis model assessment of insulin resistance (HOMA-IR) was calculated as follows: fasting blood insulin (μU/mL) × fasting blood glucose (mmol/L)/22.5. Plasma total homocysteine levels were assayed using a fluorescence polarization immunoassay (FPIA) with IMx Analyzers (Axsym Abbott Inc., Abbott Park, IL, USA) from 2009 to 2011 [22], which is an enzymatic assay using an automated chemistry analyzer Modular DPP (Roche Diagnostics, Tokyo, Japan) from 2012 to 2014, and cobas 8000 c702 from 2015 to 2019 [23].

### 2.3. Mortality Follow-Up 

The vital status of study participants was determined using nationwide death certificate data from the Korea National Statistical Office through 31 December 2019. According to the Registry for the Domestic Relations Act in Korea, all deaths in Koreans are reported to Statistics Korea, ensuring that death certificate data among Korean adults are virtually complete. To ascertain the cause of death, we used the primary underlying cause identified by the Korean National Statistical Office and classified it according to the International Classification of Diseases, Tenth Revision (ICD-10). CVD mortality was defined using the ICD-10 codes I00–I99. Cancer deaths were identified using ICD-10 codes C00–C97 as the underlying cause of death on death certificates, and dementia deaths were identified using ICD-10 codes G30 or F00 [24].

### 2.4. Statistical Analysis

The chi-square test and one-way analysis of variance were used to compare the characteristics of the study participants at baseline regarding categories of serum homocysteine level in men. Descriptive statistics summarized subject characteristics by baseline homocysteine quintiles: Q1, 1.1–8.7 µmol/L; Q2, 8.8–9.9 µmol/L (reference); Q3, 10.0–11.1 µmol/L; Q4, 11.2–12.9 µmol/L; and Q5 13.0–179.9 µmol/L. The reference category (Q2) was chosen based on previous studies that suggested that even normal-range homocysteine levels can increase the risk of dementia (≤8.9 µmol/L), and was also used as a reference for associated mortality in Korean men (<9.9 µmol/L) [9,12].

The primary endpoints were all-cause, CVD, cancer, and dementia mortality. We followed each subject from their baseline examination until the occurrence of death or until the end of 2019, whichever occurred first. Cox proportional hazards regression analyses were used to estimate hazard ratios (HRs) and 95% confidence intervals (95% CIs) for mortality outcomes. Age was used as the time scale, where subjects entered the analysis at their age at the time of their first health check-up and exited at their age on 31 December 2019, or at the date of death. 

For cause-specific mortality analyses, participants who died from other causes were censored based on their date of death. Initially, the models were adjusted using age as the time scale and then further adjusted for the study center (Seoul, Suwon), year of screening examination (in 1-year categories), smoking (never, ex-, current, or unknown), alcohol intake (<20 g/day, ≥20 g/day, or unknown), regular exercise (<3 times/week, ≥3 times/week, or unknown), BMI (continuous), and education level (less than college education, college education or higher, or unknown) (Model 1). Model 2 included additional adjustments for a history of hypertension, diabetes mellitus, medication for dyslipidemia, and eGFR (continuous). Mortality based on homocysteine levels according to multivitamin intake was estimated using the statistical methods described above. To further explore the dose–response relationship between homocysteine levels and mortality risk from all-cause, CVD, and cancer, we used restricted cubic splines with knots placed at the 5th, 27.5th, 50th, 72.5th, and 95th percentiles of the distribution within our sample. The incidence of dementia-related mortality was too low for estimation using restricted cubic splines. All analyses were performed using STATA version 16.1 (StataCorp LP, College Station, TX, USA). Statistical significance was defined as a two-sided *p*-value of <0.05.

## 3. Results

### 3.1. Characteristics of Study Subjects

Table 1 presents the baseline characteristics of the cohort population by homocysteine quintile levels, showing that homocysteine levels have a right-skewed distribution (Figure S1). Higher homocysteine levels were positively associated with older age, smoking, history of diabetes diagnosis, and systolic and diastolic blood pressures. Conversely, higher homocysteine levels were negatively associated with education level, eGFR, HDL-C level, and vitamin use.

### 3.2. Association between Homocysteine and Mortality

**Figure 2:**
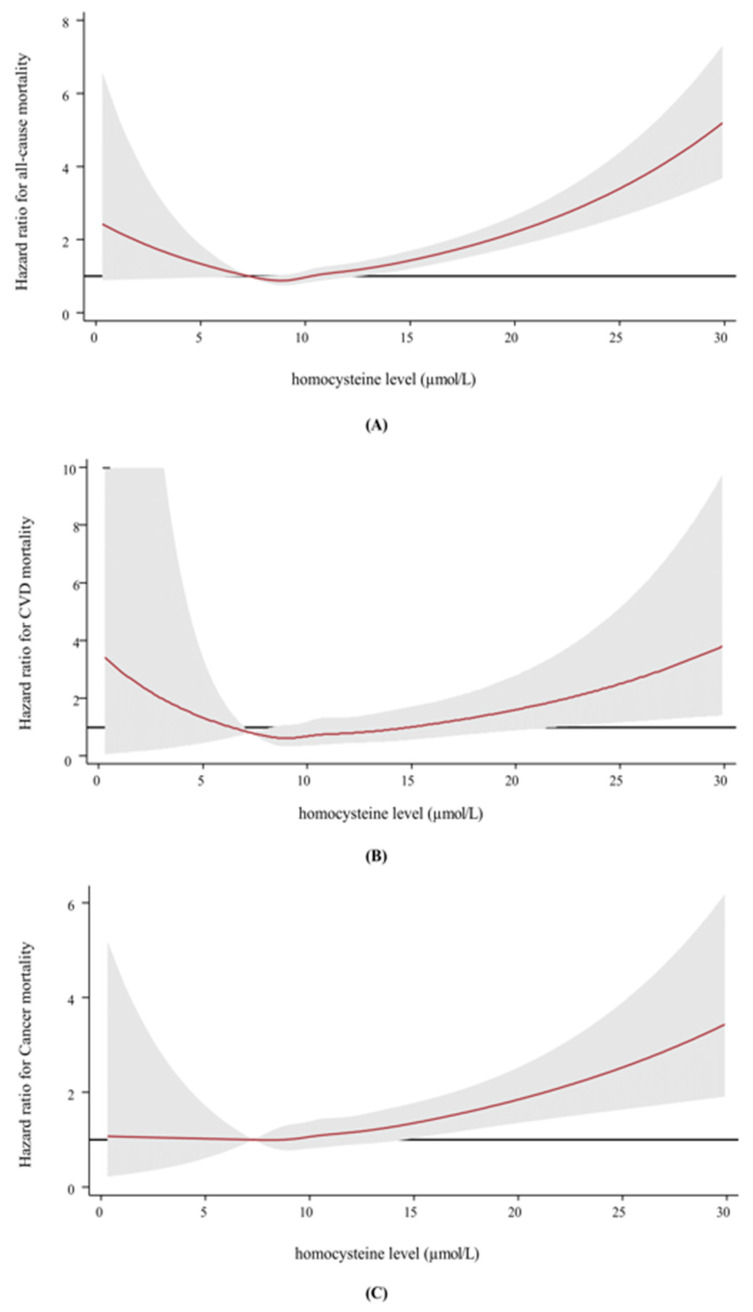
Restricted cubic splines with knots at 5th, 27.5th, 50th, 72.5th, and 95th percentiles of homocysteine level. Multivariable-adjusted hazard ratios for all-cause (**A**), cardiovascular disease (CVD) (**B**), and cancer mortality (**C**) according to total homocysteine levels among men. Red lines represent adjusted hazard ratios (with 95% confidence limits [gray zone]) for all-cause, CVD, and cancer mortality based on restricted cubic splines with knots at the 5th, 27.5th, 50th, 72.5th, and 95th percentiles of homocysteine distribution. The black lines represent the hazard ratio references. The models were adjusted for age (time scale), center, year of screening examination, smoking status, alcohol intake, physical activity, education level, body mass index, hypertension, diabetes mellitus, use of medication for hyperlipidemia, and estimated glomerular filtration rate.

During the 10-year follow-up period, a total of 10,360,349.03 person-years, and 1749 participants died, with 253 CVD deaths, 775 cancer deaths, and 11 dementia deaths. Table 2 shows the association between homocysteine levels and risk of all-cause, CVD, and cancer-related mortality. Compared with the reference category (Q2, 8.8–9.9 µmol/L), there was a significant increase in all-cause mortality rates associated with both low and high levels of homocysteine; however, this association was not observed in CVD and cancer mortality. This association remained significant even after accounting for additional covariates including education level, smoking status, history of metabolic diseases, and eGFR. The multivariable adjusted HRs (95% CI) for the reference category of homocysteine level (Q2, 8.8–9.9 µmol/L) were Q1, 1.06 (0.89–1.28); Q3, 1.15 (0.98–1.37); Q4, 1.27 (1.08–1.49); and Q5, 1.67 (1.43–1.95), respectively. In spline regression models, a U-shaped association between homocysteine levels and all-cause and CVD mortality was observed with an inflection point at 9.1 µmol/L; however, no clear U-shaped association was observed for cancer mortality (Figure 2). The HR for dementia-related deaths could not be estimated because no deaths occurred in the reference category. However, as homocysteine levels increased, the number of dementia-related deaths also increased (Table S1).

### 3.3. Subgroup Analysis: Homocysteine and Mortality Based on Vitamin Uses

Table 3 and S2 and S3 compare all-cause, CVD, and cancer mortality based on homocysteine levels between groups of vitamin supplement users and non-users. The analysis revealed a significant association between homocysteine levels and all-cause mortality in the overall cohort. However, the subgroup analysis based on vitamin use indicated that this association was absent in the vitamin-supplemented subgroup, whereas it remained significant among the subjects without supplementation. This association was not observed for CVD or cancer mortality in either of the subgroups.

## 4. Discussion

In this large-scale cohort study of healthy young and middle-aged men, it is important to note that both high and low homocysteine levels were significantly associated with increased all-cause mortality compared with the reference group (Q2, 8.8–9.9 µmol/L). This association persisted even after adjusting for baseline factors such as moderate- or vigorous-intensity physical activity, smoking status, alcohol consumption, BMI, education level, and other metabolic diseases. Notably, this study revealed a U-shaped association between serum homocysteine levels and all-cause and CVD mortality. However, the group that received vitamin supplements did not exhibit a statistically significant increase in all-cause mortality associated with homocysteine levels. These findings underscore the significance of monitoring both low and high homocysteine levels as potential risk factors for mortality in men. This finding implies that vitamin supplementation may play a protective role in mitigating this association.

Since previous studies have compared the risk of disease incidence and mortality at higher levels using the lowest homocysteine category as a reference, there are only a few studies related to low homocysteine levels [10,11,12,13]. Therefore, in this study, a large-scale sample of adult men in Korea was divided into five categories (Q1–Q5), with Q2 used as the reference category to evaluate the relationship between all-cause and cause-specific mortality (CVD, cancer, and dementia) at both low and high serum homocysteine levels. Previous studies have identified several risk factors that increase homocysteine levels, including age, lack of exercise, smoking, alcohol consumption, obesity, renal dysfunction, and metabolic diseases (hypertension, diabetes, and dyslipidemia) [14,25,26,27]. We estimated the risk of mortality by adjusting for these confounding factors and found that all-cause and CVD mortality increased at the highest homocysteine levels (≥13.0 µmol/L) and tended to increase even at the lowest levels (1.1–8.7 µmol/L) compared to the reference category (8.8–9.9 µmol/L). 

Recent studies indicate that cancer patients typically have lower average serum homocysteine levels than those without cancer, with each increase in homocysteine reducing the odds of cancer [10]. Additionally, low homocysteine levels correspond to high incidences of Alzheimer’s dementia [12], and even the lowest levels are associated with increased mortality in elderly individuals under 75 years of age with severe cognitive impairment [13]. As a result of this study, compared to the reference category, the lowest homocysteine levels were associated with increased all-cause and CVD mortality but decreased cancer mortality. Additionally, no deaths due to dementia occurred, likely because of the younger age of the subjects. Given that the study subjects were predominantly young to middle-aged men, further research that includes a broader age range is necessary. The mechanism underlying the increased risk of death when homocysteine levels are low is not fully understood, but there are several possible explanations. Low homocysteine levels may indicate poor nutritional and health statuses, potentially reflecting poor overall health [10]. In addition, lower homocysteine levels decrease DNA methylation and reduce de novo production of glutathione, which acts as an antioxidant, thereby making it more vulnerable to oxidative stress [1]. These factors may support the hypothesis that low homocysteine levels are associated with increased mortality. 

Conversely, numerous studies have investigated the association between disease incidence and mortality when homocysteine levels increase. Increased homocysteine levels are associated with higher incidences of CVD, cancer, and dementia [4,6,25,28]. Elevated homocysteine levels independently affect atherosclerosis by inducing vascular endothelial dysfunction and smooth muscle cell proliferation, which leads to CVD [29,30,31]. Previous studies that have identified a potential association between hyperhomocysteinemia and cardiovascular mortality [32,33]. Studies on older populations have shown a relationship between frailty, CVD, and homocysteine, particularly in older males [34,35]. In our study, which focuses on young and middle-aged men, we observed that hyperhomocysteinemia were associated with higher risks of cardiovascular disease (CVD) and all-cause mortality. It also causes abnormal methylation and oxidative stress in DNA, resulting in the inactivation of tumor suppressor genes and subsequent cancer development [36]. Furthermore, it is known to increase beta-amyloid production and influence the development of Alzheimer’s dementia [37]. A recent study among Koreans found that all-cause, CVD, and cancer mortality increased by 1.58, 2.23, and 1.35 times, respectively, in the highest quartile compared with the lowest quartile of homocysteine levels. Similarly, in this study, mortality rates of all-cause, cardiovascular disease, and cancer in the highest homocysteine category increased by 1.67, 1.46, and 1.44, respectively, compared to the reference category [9].

Previous studies have shown that B vitamins including folate, vitamin B6, 12 and riboflavin, which are involved in homocysteine metabolism, can reduce homocysteine levels. This reduction in homocysteine has been associated with a decreased incidence of stroke but showed no correlation with other CVD incidences or mortality from all causes and CVD [38,39,40]. Our findings indicate that all-cause mortality increased among non-users of vitamin supplements but tended to decrease among those who took vitamins. A reduction in homocysteine levels is accompanied by decreased glutathione levels and reduced antioxidant capacity [1,2]. One theory suggests that B vitamins, together with other vitamins, may serve as antioxidants instead of glutathione. However, owing to the lack of distinction between vitamin types in this study, further research is required to more thoroughly explore this theory. 

Our study had several limitations. First, we identified three causes of death: CVD, cancer, and dementia. Therefore, we did not find any association with deaths from other causes. We primarily investigated diseases previously associated with both low and high homocysteine levels, and additional research is needed to explore the mechanisms related to other diseases. Second, our study participants consisted of only Korean men, excluding women whose homocysteine levels were affected by sex hormones. However, no previous studies have reported significant findings related to homocysteine levels in women. Further research is necessary to determine menstrual cycle and hormonal levels. Third, homocysteine levels were evaluated based on a single measurement without repeated measurements and the baseline did not account for changes in serum homocysteine levels. Additional studies are warranted to consider the variability in repeated homocysteine measurements. Fourth, variations in homocysteine levels were multifactorial, extending beyond the variables adjusted in this study. These include drug use (thiazide in hypertension, fenofibrate in hyperlipidemia, metformin in diabetes, etc.), various underlying diseases (cancer, hypothyroidism, malignant anemia, atrophic gastritis, psoriasis, lupus), and genetic factors. Although we made efforts to adjust for confounding variables as thoroughly as possible, it remains possible that unmeasured or residual confounding factors exist. Finally, since our study focused on relatively healthy young Korean adult men, our findings may not be universally applicable to other demographics, including individuals with comorbidities, older age groups, and diverse ethnic backgrounds. However, the significant strengths of this study include the large sample size and use of standardized clinical, imaging, and blood test procedures. In addition, it has a strength in that it is the first study in Korea to examine the relationship between hypohomocysteinemia and mortality.

## 5. Conclusions

This large cohort study involving relatively young and middle-aged Korean adult men found that both low and high homocysteine levels increased the risk of all-cause and CVD mortality, suggesting a U-shaped association. This relationship was not statistically significant with vitamin supplementation. Further studies are needed to confirm these results by measuring homocysteine levels multiple times across different age and race groups.

## Figures and Tables

**Table 1:** Baseline characteristics of study participants by homocysteine levels among men (*n* = 221,356).

Characteristic	Homocysteine Level (µmol/L)					*p* for Trend
	Q1	Q2	Q3	Q4	Q5	
	(1.1–8.7)	(8.8–9.9)	(10.0–11.1)	(11.2–12.9)	(13.0–179.9)	
Number	41,608	43,874	44,676	46,626	44,572	
Age (years) ^1^	38.2 (9.7)	39.0 (10.0)	39.2 (10.1)	39.6 (10.5)	40.0 (11.3)	0.002
BMI (kg/m^2^) ^1^	24.5 (3.0)	24.6 (3.0)	24.7 (3.0)	24.7 (3.1)	24.6 (3.2)	<0.001
Current smoker (%)	29.8	30.5	31.4	33.3	37.5	<0.001
Alcohol intake (%) ^3^	33.1	33.8	33.8	33.2	33.8	0.450
Exercise (%) ^4^	18.5	18.8	18.3	18.5	17.5	0.005
Higher education (%) ^5^	71.2	67.0	64.2	61.4	58.0	0.009
History of diabetes (%)	5.7	5.6	5.8	6.0	6.8	<0.001
History of hypertension (%)	13.8	16.0	17.6	19.1	21.7	0.653
Medication for dyslipidemia (%)	3.2	2.8	2.8	2.8	3.5	<0.001
Use of vitamins (%)	28.6	24.3	21.7	18.7	15.1	0.033
Systolic BP (mmHg) ^1^	114.8 (11.1)	115.6 (11.4)	116.0 (11.5)	116.3 (11.8)	116.8 (12.1)	0.002
Diastolic BP (mmHg) ^1^	73.3 (8.9)	74.0 (9.0)	74.3 (9.1)	74.6 (9.2)	74.9 (9.4)	<0.001
Estimated GFR (mL/min/1.73 m^2^) ^1^	101.1 (13.2)	98.1 (13.8)	96.1 (14.1)	93.9 (14.6)	91.3 (16.2)	0.044
Glucose (mg/dL) ^1^	98.3 (17.6)	97.9 (16.8)	97.5 (16.6)	96.9 (16.2)	96.8 (16.6)	0.066
HbA1c (%) ^1^	5.6 (0.6)	5.6 (0.6)	5.6 (0.6)	5.6 (0.6)	5.6 (0.6)	0.041
HOMA-IR value ^2^	1.34 (0.88–2.00)	1.33 (0.88–1.99)	1.32 (0.86–2.00)	1.31 (0.85–2.00)	1.30 (0.84–2.00)	0.509
Total cholesterol (mg/dL) ^1^	194.9 (34.5)	196.3 (34.1)	196.9 (34.5)	197.9 (35.1)	196.9 (35.5)	<0.001
HDL-C (mg/dL) ^1^	53.3 (12.9)	53.3 (12.8)	53.1 (12.8)	53.0 (12.7)	52.9 (13.0)	0.521
LDL-C (mg/dL) ^1^	124.5 (31.6)	125.7 (31.5)	125.6 (31.9)	126.3 (32.4)	125.2 (32.9)	<0.001
Triglycerides (mg/dL) ^2^	112 (79–163)	113 (79–164)	113 (79–164)	112 (80–164)	113 (80–165)	0.006
hsCRP (mg/dL) ^2^	0.05 (0.03–0.10)	0.05 (0.03–0.10)	0.05 (0.03–0.11)	0.05 (0.03–0.11)	0.06 (0.03–0.11)	0.043

Abbreviations: BMI, body mass index; BP, blood pressure; GFR, glomerular filtration rate; HDL-C, high-density lipoprotein cholesterol; HOMA-IR, homeostatic model assessment for insulin resistance; hsCRP, high sensitivity C-reactive protein; LDL-C, low-density lipoprotein cholesterol; Data are shown as ^1^ mean ± SD or ^2^ medians (IQR) for continuous variables and *n* (%) for categorical variables. ^3^ ≥20 g of ethanol per day; ^4^ ≥3 times/week; ^5^ ≥college graduate.

**Table 2:** All-cause, cardiovascular disease, and cancer mortality according to homocysteine levels among men.

Homocysteine Level (µmol/L)	Person-Years (PY)	Number of Events	Mortality Rate (per 10^5^ PY)	Age-Adjusted HR (95% CI)	Multivariable-Adjusted HR (95% CI)	
					Model 1	Model 2
All-cause mortality						
Q1 (1.1–8.7)	1,891,440.2	216	11.4	1.11 (0.92–1.33)	1.12 (0.93–1.35)	1.06 (0.89–1.28)
Q2 (8.8–9.9)	2,034,626	243	11.9	1.00	1.00	1.00
Q3 (10.0–11.1)	2,093,202.6	311	14.9	1.13 (0.95–1.34)	1.10 (0.93–1.30)	1.15 (0.98–1.37)
Q4 (11.2–12.9)	2,211,819.1	389	17.6	1.18 (1.00–1.38)	1.14 (0.97–1.34)	1.27 (1.08–1.49)
Q5 (13.0–179.9)	2,129,261.1	590	27.7	1.49 (1.28–1.74)	1.38 (1.19–1.61)	1.67 (1.43–1.95)
*p* for trend				0.004	0.010	0.002
CVD mortality						
Q1 (1.1–8.7)	1,891,440.2	30	1.6	1.08 (0.66–1.76)	1.10 (0.67–1.79)	1.08 (0.66–1.76)
Q2 (8.8–9.9)	2,034,626	35	1.7	1.00	1.00	1.00
Q3 (10.0–11.1)	2,093,202.6	42	2.0	1.05 (0.67–1.65)	1.03 (0.65–1.61)	1.05 (0.67–1.64)
Q4 (11.2–12.9)	2,211,819.1	60	2.7	1.25 (0.82–1.89)	1.19 (0.78–1.81)	1.25 (0.82–1.90)
Q5 (13.0–179.9)	2,129,261.1	86	4.0	1.47 (0.99–2.19)	1.34 (0.90–2.00)	1.46 (0.97–2.20)
*p* for trend				0.303	0.365	0.325
Cancer mortality						
Q1 (1.1–8.7)	1,891,440.2	89	4.7	0.95 (0.72–1.25)	0.97 (0.74–1.28)	0.92 (0.70–1.21)
Q2 (8.8–9.9)	2,034,626	119	5.9	1.00	1.00	1.00
Q3 (10.0–11.1)	2,093,202.6	156	7.5	1.15 (0.90–1.46)	1.11 (0.87–1.41)	1.17 (0.92–1.49)
Q4 (11.2–12.9)	2,211,819.1	162	7.3	0.99 (0.78–1.25)	0.95 (0.75–1.21)	1.07 (0.84–1.36)
Q5 (13.0–179.9)	2,129,261.1	249	11.7	1.25 (1.00–1.55)	1.15 (0.92–1.43)	1.44 (1.15–1.80)
*p* for trend				0.621	0.691	0.422

Abbreviations: CI, confidence interval; CVD, cardiovascular disease; HR, hazard ratio (of mortality). Estimated from the Cox proportional hazards model with age as a timescale to estimate HRs and 95% CIs. The multivariable model was adjusted for age (timescale), center, year of screening examination, smoking status, alcohol consumption, regular exercise, BMI, and education level; Model 2: the same factors used in Model 1 plus an adjustment for history of hypertension, history of diabetes, and use of medication for dyslipidemia, and eGFR.

**Table 3:** Comparison of all-cause mortality based on serum homocysteine levels among men between users or non-users of vitamin supplements.

Homocysteine Level (µmol/L)	Person-Years (PY)	Number of Events	Mortality Rate (per 10^5^ PY)	Age-Adjusted HR (95% CI)	Multivariable-Adjusted HR (95% CI)	
					Model 1	Model 2
Users of vitamin supplements (*n* = 47,727)						
Q1 (1.1–8.7)	587,149.9	83	14.1	1.02 (0.75–1.37)	1.04 (0.77–1.41)	0.98 (0.72–1.32)
Q2 (8.8–9.9)	538,744.5	87	16.2	1.00	1.00	1.00
Q3 (10.0–11.1)	498,566.2	102	20.5	1.09 (0.82–1.45)	1.06 (0.79–1.41)	1.12 (0.84–1.49)
Q4 (11.2–12.9)	454,006.1	96	21.2	1.01 (0.75–1.35)	0.98 (0.73–1.31)	1.09 (0.81–1.46)
Q5 (13.0–179.9)	352,808.9	125	35.4	1.35 (1.02–1.79)	1.24 (0.94–1.65)	1.57 (1.18–2.09)
*p* for trend				0.207	0.248	0.134
Non-users of vitamin supplements (*n* = 173,629)						
Q1 (1.1–8.7)	1,304,290.3	133	10.2	1.19 (0.95–1.50)	1.21 (0.96–1.52)	1.15 (0.91–1.44)
Q2 (8.8–9.9)	1,495,881.6	156	10.4	1.00	1.00	1.00
Q3 (10.0–11.1)	1,594,636.4	209	13.1	1.15 (0.93–1.41)	1.11 (0.91–1.37)	1.17 (0.95–1.44)
Q4 (11.2–12.9)	1,757,813.1	293	16.7	1.24 (1.02–1.50)	1.19 (0.98–1.45)	1.34 (1.10–1.62)
Q5 (13.0–179.9)	1,776,452.2	465	26.2	1.49 (1.24–1.79)	1.38 (1.15–1.66)	1.67 (1.38–2.01)
*p* for trend				0.014	0.025	0.012

Abbreviations: CI, confidence interval; HR, hazard ratio (of mortality). Estimated from the Cox proportional hazards model with age as a timescale to estimate HRs and 95% CIs. The multivariable model was adjusted for age (timescale), center, year of screening examination, smoking status, alcohol consumption, regular exercise, BMI, and education level; Model 2: the same factors used in Model 1 plus an adjustment for history of hypertension, history of diabetes, and use of medication for dyslipidemia, and eGFR.

## Data Availability

The datasets generated during and/or analyzed during the current study are not publicly available due to institutional review board restrictions (the data were not collected in a manner that could be widely distributed) but the analytical methods are available from the corresponding author upon request.

## Supplementary Materials

The following supporting information can be downloaded at https://www.mdpi.com/article/10.3390/nu16162759/s1, Figure S1: Right-skewed distribution of homocysteine; Table S1: Dementia mortality according to homocysteine levels among men; Table S2: Comparison of cardiovascular disease mortality based on serum homocysteine levels among men between users or non-users of vitamin supplements; Table S3: Comparison of cancer mortality based on serum homocysteine levels among men between users or non-users of vitamin supplements.
