# Supplementary material for: Serum Homocysteine Levels and All-Cause and Cause-Specific Mortality in Korean Adult Men: A Cohort Study
Source: Nutrients. 2024 Aug 19;16(16):2759. doi: 10.3390/nu16162759 (PMC11357046; doi:10.3390/nu16162759)
Supplement: Supplementary file 1 [file nutrients-16-02759-s001.zip › nutrients-3143502-supplementary.pdf]

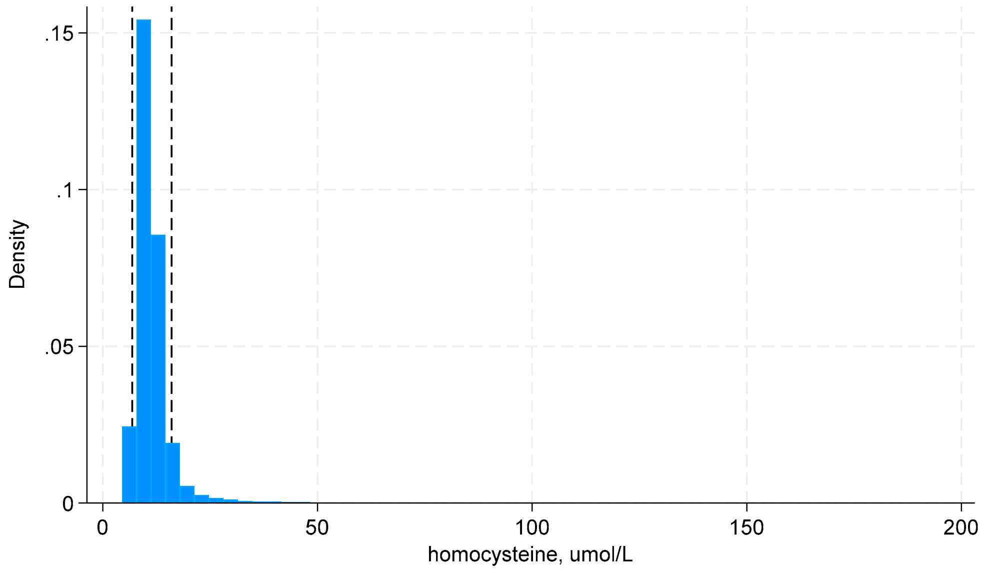

Figure S1. Right-skewed distribution of homocysteine

Table S1. Dementia mortality according to homocysteine levels among men

Homocysteine level (*μmol/L*)	Person-years (PY)	Number of events	Mortality rate (per 10^5 PY)	Age-adjusted HR (95% CI)	Multivariable-adjusted HR (95% CI)	
					Model 1	Model 2
**Dementia mortality**						
Q1 (1.1–8.7)	1,891,440.2	0	N/A	-	-	-
Q2 (8.8–9.9)	2,034,626	0	N/A	-	-	-
Q3 (10.0–11.1)	2,093,202.6	1	0.1	-	-	-
Q4 (11.2–12.9)	2,211,819.1	2	0.1	-	-	-
Q5 (13.0–179.9)	2,129,261.1	8	0.4	-	-	-
p for trend				-	-	-

Abbreviations: CI, confidence interval; HR, hazard ratio; N/A, not applicable.

During the follow-up period, no deaths due to dementia were observed in the reference group; therefore, the hazard ratio (HR) could not be estimated using the Cox proportional hazards model.

Table S2. Comparison of cardiovascular disease mortality based on serum homocysteine levels among men between users or non-users of vitamin supplements

Homocysteine level (*μmol/L*)	Person-years (PY)	Number of events	Mortality rate (per 10^5 PY)	Age-adjusted HR (95% CI)	Multivariable-adjusted HR (95% CI)	
					Model 1	Model 2
**Users of vitamin supplements (*n*=47,727)**						
Q1 (1.1–8.7)	587,149.9	10	1.7	0.89 (0.38–2.06)	0.95 (0.41–2.21)	0.95 (0.41–2.21)
Q2 (8.8–9.9)	538,744.5	12	2.2	1.00 (reference)	1.00 (reference)	1.00 (reference)
Q3 (10.0–11.1)	498,566.2	15	3.0	1.16 (0.54–2.48)	1.15 (0.54–2.46)	1.2 (0.56–2.58)
Q4 (11.2–12.9)	454,006.1	21	4.6	1.61 (0.79–3.27)	1.53 (0.75–3.12)	1.64 (0.80–3.36)
Q5 (13.0–179.9)	352,808.9	20	5.7	1.55 (0.75–3.23)	1.40 (0.67–2.91)	1.63 (0.77–3.46)
p for trend				0.822	0.803	0.926
**Non-users of vitamin supplements (*n*=173,629)**						
Q1 (1.1–8.7)	1,304,290.3	20	1.5	1.24 (0.68–2.26)	1.24 (0.68–2.27)	1.21 (0.66–2.21)
Q2 (8.8–9.9)	1,495,881.6	23	1.5	1.00 (reference)	1.00 (reference)	1.00 (reference)
Q3 (10.0–11.1)	1,594,636.4	27	1.7	1 (0.58–1.75)	0.97 (0.56–1.69)	0.99 (0.57–1.73)
Q4 (11.2–12.9)	1,757,813.1	39	2.2	1.1 (0.66–1.84)	1.06 (0.63–1.77)	1.12 (0.67–1.89)
Q5 (13.0–179.9)	1,776,452.2	66	3.7	1.39 (0.86–2.24)	1.26 (0.78–2.04)	1.36 (0.83–2.24)
p for trend				0.150	0.189	0.196

Abbreviations: CI, confidence interval; HR, hazard ratio.

Estimated from the Cox proportional hazards model with age as a timescale to estimate HRs and 95% CIs. The multivariable model was adjusted for age (timescale), center, year of screening examination, smoking status, alcohol consumption, regular exercise, BMI, education level; Model 2: the same factors used in Model 1 plus an adjustment for history of hypertension, history of diabetes, and use of medication for Dyslipidemia, and eGFR.

Table S3. Comparison of cancer mortality based on serum homocysteine levels among men between users or non-users of vitamin supplements

Homocysteine level (*μmol/L*)	Person-years (PY)	Number of events	Mortality rate (per 10^5 PY)	Age-adjusted HR (95% CI)	Multivariable-adjusted HR (95% CI)	
					Model 1	Model 2
**Users of vitamin supplements (*n*=47,727)**						
Q1 (1.1–8.7)	587,149.9	36	6.1	0.87 (0.56–1.34)	0.87 (0.56–1.36)	0.81 (0.52–1.26)
Q2 (8.8–9.9)	538,744.5	45	8.4	1.00 (reference)	1.00 (reference)	1.00 (reference)
Q3 (10.0–11.1)	498,566.2	55	11.0	1.13 (0.76–1.68)	1.08 (0.72–1.60)	1.16 (0.78–1.72)
Q4 (11.2–12.9)	454,006.1	40	8.8	0.8 (0.52–1.22)	0.77 (0.50–1.18)	0.88 (0.57–1.35)
Q5 (13.0–179.9)	352,808.9	57	16.2	1.16 (0.78–1.73)	1.07 (0.72–1.60)	1.42 (0.94–2.14)
P for trend				0.981	0.999	0.802
**Non-users of vitamin supplements (*n*=173,629)**						
Q1 (1.1–8.7)	1,304,290.3	53	4.1	1.03 (0.72–1.46)	1.06 (0.75–1.51)	1.00 (0.70–1.43)
Q2 (8.8–9.9)	1,495,881.6	74	5.0	1.00 (reference)	1.00 (reference)	1.00 (reference)
Q3 (10.0–11.1)	1,594,636.4	101	6.3	1.16 (0.86–1.56)	1.11 (0.82–1.5)	1.17 (0.87–1.58)
Q4 (11.2–12.9)	1,757,813.1	122	6.9	1.07 (0.80–1.43)	1.02 (0.76–1.36)	1.15 (0.86–1.53)
Q5 (13.0–179.9)	1,776,452.2	192	10.8	1.25 (0.95–1.63)	1.13 (0.86–1.48)	1.40 (1.06–1.85)
P for trend				0.662	0.693	0.509

Abbreviations: CI, confidence interval; HR, hazard ratio.
Estimated from the Cox proportional hazards model with age as a timescale to estimate HRs and 95% CIs. The multivariable model was adjusted for age (timescale), center, year of screening examination, smoking status, alcohol consumption, regular exercise, BMI, education level; Model 2: the same factors used in Model 1 plus an adjustment for history of hypertension, history of diabetes, and use of medication for Dyslipidemia, and eGFR.
